# Shared care management of patients with type 2 diabetes across the primary and secondary healthcare sectors: study protocol for a randomised controlled trial

**DOI:** 10.1186/s13063-016-1409-y

**Published:** 2016-06-04

**Authors:** Lene Munch, Birgitte Bennich, Anne B. Arreskov, Dorthe Overgaard, Hanne Konradsen, Filip K. Knop, Tina Vilsbøll, Michael E. Røder

**Affiliations:** Center for Diabetes Research, Gentofte Hospital, University of Copenhagen, Kildegårdsvej 28, DK-2900 Hellerup, Denmark; Institute of Nursing, Metropolitan University College, Copenhagen, Denmark; Department of Neurobiology, Care Sciences and Society, Karolinska Institutet, Hundinge, Sweden; NNF Center for Basic Metabolic Research and Department of Biomedical Sciences, Faculty of Health and Medical Sciences, University of Copenhagen, Copenhagen, Denmark

**Keywords:** Diabetes care, Diabetes complications, Randomised controlled trial, Risk stratification, Shared care, Type 2 diabetes

## Abstract

**Background:**

The prevalence of type 2 diabetes (T2D) is growing globally and hospital-based outpatient clinics are burdened with increasing numbers of patients. To ensure high quality treatment and care, it is necessary to structurally reorganise the management of patients with T2D. The objective of this study is to test if T2D patients (who are at intermediate risk of or are already having incipient diabetic complications) jointly managed by a hospital-based outpatient clinic and general practitioners (shared care programme) have a non-inferior outcome compared to an established programme in a specialised (hospital based) outpatient diabetes clinic.

**Methods:**

The study is designed as a randomised controlled trial. The shared care model will be tested during a period of 3 years, with data collection at baseline and at 12, 24 and 36 months. All patients will be offered four medical visits a year; the shared care intervention consists of one annual comprehensive check-up at the outpatient clinic and three quarterly visits at the general practitioners’ office. The control group will be followed with four quarterly visits at the outpatient clinic, including an annual comprehensive check-up. In the outpatient clinic, the patients will be treated by a specialised diabetes team, including an endocrinologist. On the basis of a predefined stratification model, we will recruit patients stratified to be at intermediate risk of or already having incipient diabetic complications. We plan to include 140 patients. The primary outcome is glycated haemoglobin. Other outcome measures include (1) the proportion of patients who meet the Danish standard indicators reflecting quality of care; (2) quality of life measured by Short Form 36; and (3) the functionality of the patients’ families measured by Family Assessment Measure III. The experiences of the patients and families when participating in the shared care program will be explored by collecting dyadic interviews.

**Discussion:**

This study will evaluate the quality of a shared care programme for patients with T2D, and provide evidence about advantages and disadvantages compared with a programme in a specialised outpatient clinic. The results may provide important information on how to organise the care for patients with T2D in the future.

**Trial Registration:**

This trial was registered with Clinicaltrials.gov on 21 October 2015, registration number: NCT02586545.

**Electronic supplementary material:**

The online version of this article (doi:10.1186/s13063-016-1409-y) contains supplementary material, which is available to authorized users.

## Background

The incidence and prevalence of type 2 diabetes (T2D) are increasing globally [1–3]. T2D is a multifactorial and chronic disease associated with serious complications and co-morbidities, and it is among the 10 leading causes of death worldwide [4, 5]. The number of adults with diabetes is estimated to rise by 55 % during a 22-year period, from 382 million individuals in 2013 to 592 million individuals in 2035 [2, 3]. Compared to the general population, patients with T2D have increased morbidity and mortality [6–8] due to an increased risk of microvascular complications and macrovascular disease [4]. Living with diabetes is also associated with major psychosocial implications and impaired quality of life [9, 10]. Furthermore, the disease causes considerable socioeconomic expenditures and healthcare costs, especially with emerging and manifest complications to the disease [3, 11, 12]. In order to prevent or delay diabetic complications, it is essential to stabilise and treat hyperglycaemia, elevated blood pressure and dyslipidaemia [13–15]. The treatment targets for these indicators are defined in evidence-based guidelines, but treatment goals are too often not achieved [16, 17]. The increasing prevalence of T2D also challenges the healthcare systems in terms of how to prioritise the limited resources. Therefore, the need for considering alternative organisational models for the management of T2D, ensuring high quality care, is urgent.

During the last decades, numerous models of diabetes management have been evaluated; among these are shared care models [18, 19]. Shared care can be defined as a joint delivery of care conducted in cooperation between general practitioners (GP) and medical specialists [20]. In most shared care models, the GP is guided in the management of T2D on the individual patient level by the specialised team [21–24]. Thus, the patient may benefit from the broad knowledge of the GP and the expertise of the medical specialists. Furthermore, there may be economic savings for the community as visits at hospital-based outpatient clinics, in general, are more expensive than GP visits. The challenges of shared care may be the difficulty in keeping track of the patients’ visiting schedule in the transition from one healthcare sector to another and potential loss of information across the interface of the primary and secondary healthcare sectors. Moreover, there is a risk of dilution of responsibility for the individual patient among the different healthcare professionals, potentially leading to ineffective decision-making and care. According to a previous Cochrane review, insufficient favourable evidence was found to support shared care programmes for patients with chronic diseases [20]. This conclusion, however, is limited by the interventions being complex and multifaceted as well as the methodological shortcomings and inadequate length of follow-up of the studies included in the review. Furthermore, the review did not test shared care programmes specifically for patients with T2D, but for chronic diseases in general.

An Australian observational study tested a community-based T2D management programme run by specially trained, primary care physicians, who were supported by an on-site endocrinologist [23]. They found a significant decrease in glycated haemoglobin (HbA_1c_) among patients in the intervention group at 12-months’ follow-up, but no change among patients in the control group. The latter consisted of patients in long-term follow-up at the outpatient clinic. However, this result may be biased because of lack of randomisation, as the intervention was not offered to all patients, but only to those who were referred by their GP to the outpatient clinic during the study period. Furthermore, the patients in the intervention group had significantly higher HbA_1c_ than the control group at baseline, but at 12-months follow-up the HbA_1c_ was similar in the two groups. In an Irish trial [24], general practice clinics were randomised to conduct either usual care or shared care to patients with T2D. Shared care consisted of educational programmes for healthcare professionals in the general practices, as well as an annual diabetes review performed in a specialised outpatient clinic. They found no significant differences in HbA_1c_ at 18-months follow-up.

Future studies testing the effectiveness of shared care interventions are recommended to be developed within research settings and also with high-quality design, well-defined interventions, and with an extended length of follow-up period [20]. According to Danish regional guidelines, patients with T2D in intermediate risk for progress of diabetic complications, identified via a risk stratification model, are recommended to be followed in tight collaboration between GP and specialised and hospital-based outpatient clinics [25]. In line with these recommendations, we plan a randomised controlled trial to test a shared care model in a well-defined group of patients with T2D, with well-defined transition and decision making across the healthcare sectors.

## Methods/Design

### Study design

**Fig. 1:**
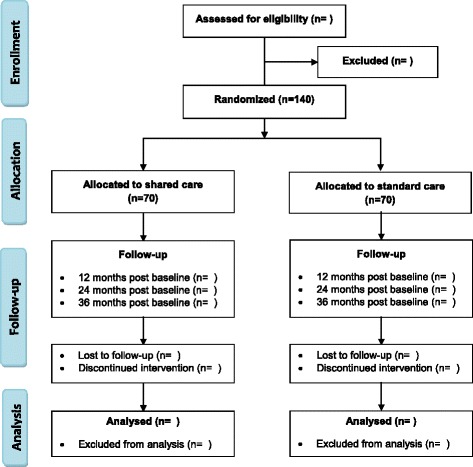
Study flow diagram. The planned flow of participants through the different stages of the study

The present trial is a proof-of-concept study. We aim to test the effect of a new model of organisation and management of diabetes care, and compare it with a standardised care management programme in a specialised outpatient clinic. The study is designed as a randomised controlled trial testing for non-inferiority between the two programmes. We hypothesise that patients with T2D followed in a shared care programme (intervention) will have a comparable primary outcome (HbA_1c_) compared to patients receiving standard care (control). The study flow is indicated in Fig. 1. The study follows the guidelines of Standard Protocol Items: Recommendations for Interventional Trials (SPIRIT) (Additional file 1).

### The settings

The study is a cross-sectional study with several settings. The intervention takes place in the diabetes outpatient clinic at Gentofte Hospital, University of Copenhagen, Hellerup, Denmark, as well as in a number of general practices in the Capital Region of Denmark. The control group is managed solely in the outpatient clinic at Gentofte Hospital placed in northern Copenhagen, Denmark. Gentofte Hospital covers four municipalities with a total population of approximately 224,000 inhabitants, and with approximately 10,000 inhabitants diagnosed with diabetes [22, 23]. There are 106 general practices located in the four municipalities with a total of 150 GPs. The hospital-based outpatient clinic is staffed by a diabetes team consisting of endocrinologists with special interest in diabetes, diabetes nurses, a dietician, podiatrists, and trained nurses who perform fundus photography, which are evaluated by ophthalmologists. Currently, the diabetes team is responsible for the care of approximately 1200 patients with T2D.

### Participants

**Fig. 2:**
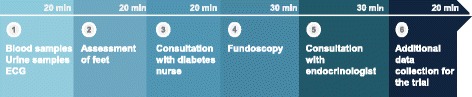
Content of the annual comprehensive check-up in the outpatient clinic

We will recruit eligible patients followed for T2D by either their GP or at the outpatient clinic. A patient is only eligible if their GP accepts participation in the study. Therefore, we will recruit GPs before recruiting study participants. Invitations to participate will be distributed by mail to all regional GPs referring patients to Gentofte Hospital. We intend to include approximately 25 GPs, aiming at recruiting six patients from each practice or until we have reached the predefined number of patients in the study (n = 140). An endocrinologist and the primary investigator will make appointments for visiting GPs who have accepted to participate. The duration of the individual visit at the GP’s office will be of approximately 2 hours. During the visit, we will identify patients who are eligible to participate according to the inclusion criteria. Patients managed by the GP and patients previously referred from the individual GP to be managed at the outpatient clinic are identified. Thus, both patients who have their T2D managed solely by their GP, or at the outpatient clinic, will be recruited. Eligible patients will be contacted by phone and, if the patient shows interest in participating in the study, written information will be sent by regular mail or e-mail. If the patient agrees to participate in the study by giving written consent, she/he will be invited to a baseline-screening visit in the outpatient clinic (Fig. 2). If all inclusion criteria are fulfilled and none of the exclusion criteria are met, patients will then be randomised to one of the two groups. Patients who decline to participate or do not meet the inclusion criteria will continue their usual T2D care either at the GP or at the outpatient clinic.

### Inclusion criteria

Patients with T2D over the age of 18 years, able to understand, speak and write Danish, willing to give oral and written consent, and at predefined risk stratification level 2, are eligible for the study. According to the risk stratification model applied (Table 1), patients with T2D can be stratified into three levels: level 1 (uncomplicated), level 2 (intermediate risk) or level 3 (high risk). The model is an organisational tool intended to graduate the medical care and treatment according to the severity and complexity of T2D. Patients stratified at level 1 have well-controlled T2D and are recommended to be followed in general practice, whereas patients at level 3 are recommended to be followed in hospital-based outpatient clinics, as they have dysregulated T2D and/or increasing severity of complications and/or co-morbidity. Patients stratified at level 2 are moderately dysregulated T2D and/or having incipient complications. These patients are recommended to be followed in closer collaboration between hospital and general practice. The model was originally defined in a report from the Capital Region of Denmark compiled by diabetes specialists, other experts and GPs [26] and based on current evidence on treatment goals and cut-off points in diabetes management [27–30]. In the present trial, the model is slightly modified to make the model more operational. In a recent study of the population in our outpatient clinic, we found that 54 % of the patients were stratified to level 2, while less than 5 % were stratified to level 1 [31].

**Table 1:** Risk stratification model for patients with type 2 diabetes

	Level 1	Level 2	Level 3
HbA_1c_ (mmol/mol)	<53	53–75	>75
Blood pressure, systolic/diastolic (mm Hg)	<130/80	130/80–160/90	>160/90
Metabolic complications	No	Severe insulin resistance^a^	Very fluctuating plasma glucose^b^ or severe hypoglycaemia
CVD^d^	No	One previous MACE	>1 MACE, symptomatic CVD or NYHA II-IV
Diabetic foot disease	No	Peripheral neuropathy^c^ or artery disease^d^	Previous or existing ulcer or Charcot foot
Retinopathy	No or simplex retinopathy	Progression of retinopathy	Macula oedema or proliferative retinopathy
Nephropathy	No	Micro-albuminuria^e^	Macro-albuminuria^f^

All parameters in level 1 have to be fulfilled to be allocated to risk stratification level 1. At risk stratification level 2, at least one parameter has to be fulfilled in level 2, and none in level 3. Patients at level 3 have to fulfil at least one of the parameters in level 3 [16].

*HbA*_*1c*_ haemoglobin A_1c_, *CVD* cardiovascular disease, *MACE* major cardiovascular event, *NYHA* the New York Heart Association functional classification in patients with heart disease [36]

^a^Severe insulin resistance: Insulin dose > 2.0 U/kg/day

^b^Very fluctuating plasma glucose: Daily plasma glucose values of > 15 mmol/L or < 5 mmol/L

^c^Peripheral neuropathy: Vibration perception threshold ≥ 25 mV evaluated by a biothesiometer

^d^Peripheral artery disease: Ankle-brachial index < 0.9 with or without symptomatic claudication

^e^Micro-albuminuria: > 1 occasion of urine albumin/creatinine ratio between 30 and 299 mg/g

^f^Macro-albuminuria: Urine albumin/creatinine ratio ≥ 300 mg/g or an estimated glomerular filtration rate < 45 mL/min

### Exclusion criteria

Patients are excluded if they have other types of diabetes than T2D, are at risk stratification level 1 or 3 (Table 1), are pregnant or breastfeeding, have severe co-morbidity with a life expectancy less than 5 years, or are under such conditions that they will not be able to attend or complete the appointments (e.g. due to dementia or psychiatric conditions). Furthermore, we will exclude patients who are either participating in other studies that potentially may affect the primary outcome of our study, or patients participating in studies which include blood sampling amounting to > 5 % of the blood volume in a 2 month period prior to the randomisation and/or the follow-up visits. The risk stratification level is assessed by the endocrinologist during the 12- and 24-months follow-up. If the patient has shifted from level 2 to level 3, the patient is excluded from the study. These patients will be offered long-term follow-up in the diabetes outpatient clinic. Patients changing from level 2 to level 1 will continue their participation in the study.

### Randomisation

Patients will be randomised to the intervention or control groups in a 1:1 ratio. The randomisation sequence is computer generated by a statistician, who is not participating in the study. A secretary in the outpatient clinic will handle the script and instructions. The secretary will prepare sealed envelopes in accordance with instructions, and the envelopes will be stored and administered by the secretary during the period of inclusion. There will be an envelope prepared for each GP, and we will randomise the patients in blocks of two since we aim at each GP having an equal number of patients in the intervention and control group. The randomisation will be performed immediately after the baseline screening visit. Healthcare professionals involved in the study, including the principal investigator, will be blinded to the randomisation process. The allocation will be revealed after the screening visit. Further blinding is not possible due to the circumstances of the intervention.

### Intervention group

**Fig. 3:**
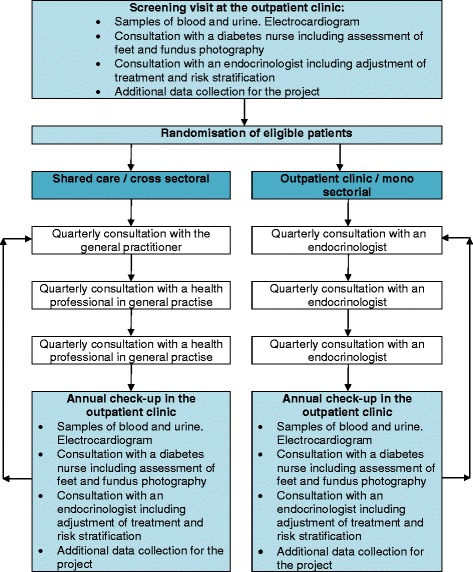
Schematic diagram of the intervention

Patients in the intervention group will be randomised to a shared care programme with joint participation in the delivery of care by the GP and the outpatient clinic. It comprises four visits a year, namely one annual comprehensive check-up at the outpatient clinic and three quarterly visits at their GP (Fig. 3). The annual comprehensive check-up will include biochemical tests, electrocardiogram, fundus photography, feet examination including biothesiometry to detect early signs of peripheral neuropathy, consultations with a diabetes nurse focusing on life-style intervention and compliance, and an endocrinologist focusing on an individualised optimal pharmacologic treatment, prevention of complications and a target-oriented treatment plan for the following year (Fig. 2). All the procedures will be performed the same day during a two-to-three hour visit. The first visit at the GP’s office after the annual comprehensive check-up will be carried out by the GP. The following two visits can be handled by either the GP or the practice nurse according to standard procedures at the local primary healthcare clinic. No further interventions related to the trial will be conducted in the general practices, as healthcare professionals should manage the delivery of care as usual. In order to secure transition of knowledge from the outpatient clinic to the GP, a copy of the medical record statement will be sent electronically to the GP after the annual comprehensive check-up. This report includes a plan and targets for the T2D treatment on different key parameters such as HbA_1c_ and blood pressure. Furthermore, we will use a direct electronic communication system to enable communication across the interface of the different GPs and the outpatient clinic. This is a well-established secure communication channel for exchanging messages, which is available for all healthcare professionals in both the primary and secondary healthcare sector in the Capital region of Denmark. In case of urgent problems or questions, the GPs can call a Diabetes Hotline, which can be called every day during working hours and is answered directly by an endocrinologist in the outpatient clinic. Patients in the intervention group will receive a leaflet and verbal information about the free lifestyle rehabilitation services offered by their local municipality. This includes patient education, physical activity, dietician consultations, and smoking cessation courses. Furthermore, they will be informed that questions concerning T2D, in the period between the annual comprehensive check-ups, should be addressed to the health professionals at the GP’s office.

### Control group

Patients in the control group will be attending a well-established visiting algorithm consisting of four visits a year with the diabetes team at the outpatient clinic, namely an annual comprehensive check-up, identical to the one received by the intervention group, and three quarterly visits. During the three quarterly visits, they will be seen by an endocrinologist according to the visiting algorithm (Fig. 3). Patients receiving usual care will be offered lifestyle rehabilitation services such as patient education, physical activity and dietician consultations at the hospital. Any questions or uncertainties concerning their T2D should be addressed to the healthcare professionals at the outpatient clinic.

### Study data

All study data will be collected in connection with the annual comprehensive check-up in the outpatient clinic at the baseline-screening visit, as well as 12, 24 and 36 months after baseline. Data will be obtained from medical records, laboratory databases, self-reported patient questionnaires and face-to-face by the primary investigator who does not take part in the treatment or care of the patients. All data will be registered in an Epidata database [32]. The database is stored at Gentofte Hospital in a secure purpose-designed password-protected web-based file system.

The primary outcome is the difference in mean HbA_1c_ between the two groups measured at 12-, 24- and 36-months follow-up. The secondary outcome is the proportion of patients who meet the set of Danish standard indicators reflecting quality of care at 12, 24 and 36 months follow-up [33]. These indicators cover seven areas, including HbA_1c_, blood pressure, low density lipoprotein, albuminuria, fundus photography, foot examination, smoking status and proper treatment and control of these factors. The indicators are primarily based on process variables, but do include a few numerical variables.

Other outcomes are listed below:

*Clinical characteristics:* Blood pressure, weight and body mass index.

*Diabetes-related laboratory parameters and characteristics:* C-peptide, insulin, lipids, liver function tests, kidney function tests, electrolytes, coagulation factors, thyroid-stimulating hormone, cardiovascular biomarkers, vitamin D, urine albumin/creatinine ratio, and registration of diabetes-related medication.

*Healthcare professionals’ experiences with shared care:* A questionnaire evaluating the experiences of the participating GPs and the endocrinologists after the completion of the 12- and 36-month follow-up periods.

*Patient-reported outcomes:* Estimation of general quality of life, diabetes-related symptoms, alcohol consumption patterns as well as patients’ experiences of family network and function.

The four questionnaires used are:

*Short Form 36 (SF-36):* SF-36 is a generic self-administered patient questionnaire measuring quality of life [34, 35]. The 36 items of SF-36 are grouped into eight domains: physical function, physical limitations, bodily pain, general health, vitality, social function, emotional limitation and mental health. The answers of each scale are transformed into a 0–100 score. An increase in the score indicates less disability. The eight domains can be divided into physical and mental health summary scales, respectively. SF-36 has been validated and is reliable in a range of languages, including Danish [36].

*Diabetes Symptom Checklist – Revised (DSC-R):* DSC-R is a disease-specific questionnaire measuring the occurrence and perceived burden of T2D-symptoms [37]. It comprises 34 items grouped into eight domains: hyperglycaemic, hypoglycaemic, psychological-cognitive, psychological-fatigue, cardiovascular, neurological-pain, neurological-sensory, and ophthalmologic [37, 38]. It is a self-administered questionnaire. For each item, patients answer whether or not they have experienced the described symptom within the last 4 weeks, and if “yes”, they specify how troublesome the symptom has been on a 5-point Likert scale. Each domain is calculated by adding up the scores for each item (range, 1–5 points) and then divide the summed score by the number of items within the domain. A reduction in score indicates less diabetes symptom distress. DSC-R is a previously validated, reliable measurement instrument, sensitive to changes over time [38].

*The Alcohol Use Disorders Identification Test (AUDIT):* The AUDIT was developed on behalf of the World Health Organization as a screening tool for identifying persons with hazardous and harmful alcohol consumption patterns [39, 40]. It consists of 10 questions divided into three domains: hazardous alcohol use, dependence symptoms and harmful alcohol use. Each question has three or five response options and each answer triggers a score of 0–4 points. The scores are added up to a total score ranging from 0 to 40 points. Seven points is defined as the cut-off representing an alcohol problem. A higher score indicates a more hazardous and harmful alcohol consumption pattern [39].

*Family assessment measure III (FAM-III):* FAM-III is a self-administered questionnaire assessing family functioning. It consists of three 14-item questionnaires; the general scale examines the general health of the family; the dyadic relationship scale assesses how family members view their relationship with each other; and in the self-rating scale, individuals rate their own functioning within the family. Each item is answered on a 4-point Likert scale, giving a total score of 0–168. Low scores indicate a strong and well-functioning family, while high scores indicate a problematic and dysfunctional family. It is a useful, validated instrument for measuring family functioning during the course of treatment [41].

### Population size calculation

A power calculation was conducted according to criteria for non-inferiority trials [42, 43], and based on the primary outcome measure Hb_A1c_ at 12-month follow-up. The non-inferiority margin was set at 0.4 % (1 % is approximately 11 mmol/mol) based on both clinical judgement and a guideline from the European Medicines Agency identifying the non-inferiority margin for HbA_1c_ above 0.3 % (3 mmol/mol) [44]. The standard deviation of HbA_1c_ was set at 10 mmol/mol, based on a recent study on patients with risk stratification level 2 attending our outpatient clinic [31]. With a non-inferiority margin at HbA_1c_ 4.4 mmol/mol, an alpha of 0.05 and a power at 0.8, as well as an estimated dropout rate of 10 %, the sample size has been estimated to 140 participants.

### Qualitative study data

In the qualitative process analysis of the study the aim is to explore and explain how the specific programme works for those involved, rather than focusing on endpoints [45]. Data will be generated by interviews with patients and family members 1 year after inclusion in the study. Dyadic interview will examine the interaction between the participants, and thereby obtain details of discrepancies and similarities between them [46].

Interviews will initially be guided by a semi-structured interview guide developed on the basis of knowledge from earlier studies and the FAM-III questionnaire. The interview guide will change dynamically according to theoretical sampling and therefore data collection and analysis of previous interviews will guide the following interview in a constant comparative method throughout the entire research process [47]. Approximately 20 patients (10 from each arm) and their family members will be included consistent with theoretical sampling and hence preliminary inclusion criteria’s are not desirable nor possible. Further participants can be included if theoretical saturation is not achieved [47]. All interviews will be conducted at the patient’s home or at the hospital according to the patient and the family member’s wish. Participants will be excluded if they for any reason are not able to manage a 45–60 minute interview.

### Statistical analysis

We will present and compare the baseline demographics as well as clinical and diabetes-related characteristics of the intervention and the control group. The statistical comparison will be conducted on the basis of *t* tests or analysis of variance as appropriate for comparing means and χ^2^ tests for categorical or binary data. The primary outcome will be tested by constructing a 90 % two-sided confidence interval for the difference of the means of HbA_1c_ between the intervention and the control group. We use the lower bound to determine non-inferiority. Other outcomes will be tested with superiority analyses such as linear or logistic regression analysis. For observed differences we will consider a *P* value less than 0.05 as statistically significant.

### Qualitative data analysis

The interviews will be audio-taped for verbatim transcription. The transcripts will be analysed according to the principles of grounded theory [47, 48] to identify concepts, core categories and patterns. The computer software program NVivo10 will be used to support organising data [49]. With a main focus on “what is going on in the data” tentative links between categories will emerge and pattern out theoretical connections, constitute the core category, and further theory building. In order to increase validity, all steps during analysis will be discussed with researchers experienced in grounded theory. Finally, all authors will determine whether findings have fit, work, and are relevant and modifiable [47].

## Discussion

The overall objective of this study is to evaluate whether sharing general and specialised healthcare is comparable to the specialised care delivered in a hospital-based outpatient setting for patients with T2D who are at intermediate risk of or are already having incipient diabetic complications. The hypothesis is that the changes in HbA_1c_ at 12-, 24- and 36-month follow-up are comparable between the intervention and the control group. According to previous studies the evidence for shared care in different chronic diseases, including T2D, is not clear [20]. Testing new shared care models as a way of alternative organisation of care for patients with T2D is therefore important. The majority of studies on shared care for patients with T2D have been conducted 15–20 years ago. However, the increasing number of patients with T2D who are referred to the outpatient clinics as well as the development and improvements of electronic systems, clinical databases and communication channels during the last decades has made it highly relevant to test shared care models in new settings. Shared databases and electronic correspondence across the interface of the primary and secondary healthcare sectors are expected to blur the boundaries between the different healthcare sectors.

If shared care is shown to be effective, it can provide new ways of organising care for the globally increasing number of patients with T2D, allowing more patients to benefit from the specialised diabetes team at outpatient clinics while keeping a close contact with the GP, who often has a broader knowledge of the individual. A well-structured clinical follow-up and treatment, supported by the diabetes team, can potentially improve the long-term prognoses for patients who are at intermediate risk of or already having incipient diabetic complications. The Steno-2 study showed that intensified focus on lifestyle and multi-pharmacological treatment of patients with T2D and microalbuminuria reduced the development of micro- and macrovascular complications significantly at 13.3 years follow-up compared to patients receiving conventional therapy by the GP [13]. Preventing or delaying the onset of complications for patients with T2D is essential, as the development of these complications significantly increase socioeconomic costs and can cause personal disabilities. Thus, it is well known that, as the patient moves from a state corresponding to a risk stratification level 2 to level 3, it is associated with multiple increases in direct and indirect cost to the healthcare sector and the community [11, 12].

Multiple parameters are known to have a significant effect on outcome. Apart from testing the primary outcome, HbA_1c_, we plan to investigate factors such as quality of life, blood pressure, lipid levels, family function and other clinical characteristics in order to test any negative or positive impact of shared care in accordance with previous studies showing a prognostic impact of these factors, e.g. blood pressure [50] and family network and functionality [51, 52].

The strength of our study is that it is designed as a randomised controlled trial. The majority of previous studies, which have tested shared care models for patients with chronic diseases, have been with limited robustness in study designs in terms of testing multifaceted interventions, limited periods of follow-up and not meeting the quality criteria of Cochrane Review Group for randomised controlled trials [20]. Furthermore, the qualitative process evaluation can provide knowledge about the appropriateness of disseminating a particular intervention to other relevant sites and about which elements to improve and which to keep [53].

Since the study is conducted in a Danish setting, the results may not be generalised to healthcare settings in other countries. However, we believe that the results may inspire healthcare planners in other countries, as the organisation of the Danish healthcare system in a primary and secondary healthcare sector is comparable to the organisation of healthcare in a range of other countries in Europe and elsewhere.

Other limitations must also be considered. A potential bias in the study is that we recruit GPs who have shown interest in the project, and therefore may not be representative for the whole population of GPs. It could therefore be either GPs who have a special interest and knowledge about T2D or GPs who want to learn and become more knowledgeable about T2D. However, the substantial number of different GPs in the present trial may result in variability in the GPs interest in and knowledge about T2D. We aim to include approximately six patients from each GP, avoiding a given GP from including significantly more patients than the others.

If the findings of this study support future implementation of a shared care model for patients with T2D, more patients could benefit of the specialised diabetes team, but with fewer patients followed solely on a routine basis in the outpatient clinic, thus potentially increasing cost–benefit relationships. Both patients and the GPs may benefit from tight and improved collaboration with the diabetes team. The trial may therefore impact future organisation of diabetes care.

### Trial status

The trial is ongoing. The recruitment of patients started August 2015 and is expected to be completed by December 2016.

## Abbreviations

AUDIT, The Alcohol Use Disorders Identification Test; DSC-R, Diabetes Symptom Checklist – Revised; GP, General Practitioner; FAM-III, Family assessment measure III; Hb_A1c_, glycated haemoglobin; SF-36, Short Form 36; T2D, Type 2 diabetes.

## Additional file

Additional file 1: SPIRIT 2013 Checklist: Recommended items to address in a clinical trial protocol and related documents. (DOC 120 kb)
